# Feeding Preferences of Abyssal Macrofauna Inferred from *In Situ* Pulse Chase Experiments

**DOI:** 10.1371/journal.pone.0080510

**Published:** 2013-11-26

**Authors:** Rachel M. Jeffreys, Ciara Burke, Alan J. Jamieson, Bhavani E. Narayanaswamy, Henry A. Ruhl, Kenneth L. Smith, Ursula Witte

**Affiliations:** 1 School of Environmental Sciences, University of Liverpool, Liverpool, Merseyside, United Kingdom; 2 Oceanlab, University of Aberdeen, Newburgh, Aberdeenshire, United Kingdom; 3 Ecology Department, Scottish Association for Marine Science, Scottish Marine Institute Oban, Argyll, United Kingdom; 4 National Oceanography Centre, University of Southampton Waterfront Campus, Southampton, Hampshire, United Kingdom; 5 Monterey Bay Aquarium Research Institute, Moss Landing, California, United States of America; University of Southampton, United Kingdom

## Abstract

Climatic fluctuations may significantly alter the taxonomic and biochemical composition of phytoplankton blooms and subsequently phytodetritus, the food source for the majority of deep-sea communities. To examine the response of abyssal benthic communities to different food resources we simulated a food sedimentation event containing diatoms and coccolithophorids at Station M in the NE Pacific. In one set of experiments we measured incorporation of _diatom_C and _cocco_N into the macrofauna using isotopically enriched ^13^C-diatoms and ^15^N-coccolithophores. In a second experiment we measured incorporation of C and N from dual-labelled (^13^C and ^15^N) diatoms. The second experiment was repeated 2 months later to assess the effect of seasonality. The simulated food pulses represented additions of 650 – 800 mg C m^−2^ and 120 mg N m^−2^ to the seafloor. In all cases rapid incorporation of tracer was observed within 4 days, with between 20% and 52% of the macrofauna displaying evidence of enrichment. However, incorporation levels of both _diatom_C and _cocco_N were low (<0.05% and 0.005% of the added C and N). Incorporation of labelled diatoms was similar during both June and September suggesting that the community was not food limited during either period. We found no evidence for selective ingestion of the different food types in the metazoan fauna suggesting that macrofauna do not have strong preferences for diatom vs. coccolithophore dominated phytodetrital pulses. C∶N ratios from both experiments suggest that the metazoan macrofauna community appear to have higher C demands and/or assimilation efficiencies compared to N. Concomitantly, the foraminifera preferentially selected for _diatom_N over _cocco_N, and we suggest that this may be related to foraminiferal requirements for intracellular nitrate. These experiments provide evidence that abyssal faunal feeding strategies are in part driven by an organism's internal stoichiometric budgets and biochemical requirements.

## Introduction

The abyssal seafloor is a vast ecosystem covering ∼54% of the Earth's surface [1]. Abyssal communities are energy limited and rely on the input of particulate organic matter (POM) produced through photosynthesis in the surface waters for food [2], [3]. A portion of the flux of POM is deposited at the seafloor as large aggregates of intact phytoplankton cells known as phytodetritus [4], [5], and a tight coupling between this external food source and abyssal benthic community structure and functioning has been documented [6]–[8]. Recent time-series data from two abyssal observatories have revealed that climate-driven variations in food supply in terms of its timing and magnitude, has resulted in broad changes in both community structure and ecosystem functioning [9]–[11].

Energy limitation at abyssal depths (>4000 m) has led to a reduced standing stock of macrofaunal organisms when compared to smaller size classes i.e. bacteria and meiofauna [12]. In spite of this the macrofauna are an important facet of abyssal communities. Macrofauna can influence organic matter processing, burial and nutrient cycles through feeding processes [13], [14], bioturbation [15], [16] and remineralization [17]. More recently macrofaunal activity (e.g. grazing and microhabitat destruction) has been linked to lower incorporation of labelled carbon by bacteria [18], [19].

Given the connectedness of climate to deep sea systems and that macrofaunal communities are known to be sensitive to changes in food supply [7], [9], [20], the importance of understanding feeding preferences/resource selection and the consequences for biogeochemical processes in the deep sea is increasing. Macrofauna respond within 36 hours to phytodetritus deposition by ingestion and bioturbation [21]–[24]. Pulse chase experiments have demonstrated that macrofauna can dominate the incorporation of labelled carbon [25]–[27]. These experiments also show differential utilization of phytodetritus and nutrients between macrofaunal taxa [21], [28]–[30]. Similarly, lipid distributions of deep-sea polychaetes show selective ingestion of fatty acids and sterols indicative of microalgae [31], [32]. In shallower waters, pulse-chase experiments have shown that macrofauna are capable of selecting for a particular type of microalgae, i.e. ice algae over phytoplankton [33], [34]. In the deep sea little is known about macrofaunal resource selection. While macrofauna have been shown to have long-term variations in functional group abundances in polychaetes, for example, the links to food supply remain unclear [35].

The long-term abyssal monitoring site ‘Station M’ in the NE Pacific provides a good setting to study macrofaunal resource selection. Benthopelagic coupling has been studied in detail at Station M since 1989 [10], [36]. The macrofauna are known to respond to pulses of phytodetritus and climate-driven changes in food supply [9], [20], [30]. This study aims to test the hypotheses: (1) that macrofauna will preferentially select for a particular type of phytodetritus (2) uptake of simulated phytodetrital pulses varies seasonally and, (3) that processing of phytodetritus by macrofauna will be dictated by internal biochemical (i.e. C and N) demands. The results are discussed in the context of macrofaunal community and food web structure.

## Materials and Methods

### Study site

Station M (34°50′ N, 123°00′ W) is located at a water depth of ca. 4100 m in the Northeast Pacific. This site is located at the base of the Monterey deep-sea fan, ∼220 km west of Point Conception, California. An overview of the biology, chemistry and oceanography of Station M are given in Smith and Druffel [37]. Fluxes of particulate organic carbon (POC) and particulate total nitrogen (PTN) are generally highest during the spring and autumn months, reaching up to 25 mg C m^−2^ d^−1^ and 3.4 mg N m^−2^ d^−1^, respectively (50 metres above bottom, m.a.b.) and display high inter-annual variability [36], [38], [39]. Detrital aggregates are present at the seafloor from June to December, with highest occurrences observed during the months of September and October [40]. Detrital aggregate organic carbon (OC) at the seafloor can reach peaks of 161 mg C m^−2^ with aggregates having OC and TN values ranging from 5 to 110 mg OC g^−1^ and 2 to 13 mg N g^−1^, respectively [40], [41]. This study took place within the US EEZ on the US *RV Western Flyer* cruise PULSE 52 (5^th^–10^th^ June 2007) and PULSE 53 (17^th^–24^th^ September 2007). The experiments and sampling were conducted as part of a routine sampling event and no specific permission was needed for this sampling. The experiments and sampling did not involve vertebrates or any endangered/protected species.

### Cultivation of labelled food sources

The centric diatoms *Thalassiosira weissflogii*, *Chaetoceros mullerri* and *Skeletonema costatum* (Coscinodiscophyceae) and the coccolithophore *Emiliania huxleyi* (Prymnesiophyceae) were chosen as food sources as they have been observed both in the California current spring bloom and within detrital aggregates sampled at the seafloor at Station M [42], [43]. Algae were cultured in artificial seawater using L1 medium [44] at 16°C (light∶dark = 16∶8; salinity  = 35; pH = 7.8 to 8.2; duration  = 21 days; [45]. The artificial seawater medium inoculating the *T. weissflogii, C. mullerri* and *S. costatum* was amended with ^13^C-bicarbonate (99% atom % enriched NaH^13^CO_3_, Cambridge Isotope Laboratories). Similarly, the medium inoculating *E. huxleyi* and *S. costatum* was amended with ^15^N-sodium nitrate (98% atom enriched Na^15^NO_3_). Algae were harvested by centrifugation (1500 rpm at 16°C for 15 mins) and washed 3 times in an isotonic solution to remove excess label. Algae were flash frozen using liquid nitrogen, N_2(*l*)_ to reduce cell damage and then lypholized. Cell sizes and the biochemical composition of the amended food sources are given in Table 1. Three different diatom species were used in these experiments, which may present difficulty in comparing experimental data. However, the biochemical composition of these three genera, are known to be similar [46] and we feel comparisons between diatoms (including the three species used) and coccolithophorids are justified.

**Table 1:** Biochemical composition of algal cultures used in this study.

Species	Cell Size (µm)	C (%)	^13^C (atom %)	N (%)	^15^N (atom %)	C:N
*Chaetoceros muelleri*	4–10	11.1	14.8	2.0	0.4	5.6
*Emiliania huxleyi*	4–10	7.7	1.1	0.9	7.0	8.5
*Skeletonema costatum*	4–10	13.8	21.7	2.6	4.6	5.3
*Thalassiosira weissflogii*	6–10	19.8	48.8	n.d.	n.d.	n.d.

### Experimental design

Isotopically labelled food choice experiments were conducted in situ using a prototype of the Oceanlab spreader mesocosms [19], [21]. Each spreader consisted of a transparent acrylic tube (diameter: 29 cm, height 50 cm) with a centrally fixed cartridge of isotopically labelled food sources, which is released by depressing an elastically tensioned plunger. Spreaders were deployed by the ROV *Tiburon*, releasing known doses of isotopically labelled phytodetritus onto replicate 0.066 m^−2^ areas of the seafloor.

During June 2007 three spreaders were deployed containing a slurry of *C. mulleri* and *E. huxleyi* representing an addition of 0.8 g C m^−2^ and 0.12 g N m^−2^, equivalent to ∼40% and 58% of the annual POC and PTN flux, at the seafloor (Experiment 1). The fourth spreader was deployed containing the dual labelled diatom *S. costatum* slurry, here an addition of 0.7 g C m^−2^ and 0.12 g N m^−2^, equivalent to ∼35% and 58% of the annual POC and PTN flux, respectively was applied to the seafloor (Experiment 2). Following this during September 2007 four replicate spreaders were deployed containing a suspension of the diatom *T. weissflogii*, representing an addition of 0.65 g C m^−2^, equivalent to 34% of the annual POC flux at the seafloor.

Spreaders were deployed on undisturbed areas of the seafloor and after any resuspended sediment had settled the experiments commenced. Experiments were terminated after four days and sub-sampled using 70 mm diameter push cores recovered by the ROV. Three pushcores from each spreader were retrieved in June and a single core from each spreader in September. Cores for macrofaunal analyses were sectioned 1 cm intervals to 5 cm in June and at 0–2 cm and 2–5 cm in September. In order to compare data between seasons we combined data obtained (abundance, biomass, label incorporation) from the 1 cm intervals in June to 0–2 cm and 2–5 cm for comparison with September. Background/control push cores were taken close to the spreaders at the start of the experiments to provide natural stable isotope values of the macrofauna and sediments (n = 8 in June and n = 3 for September).

### Sampling procedures and isotope analyses

Sectioned cores were wet-sieved through a 250 mm mesh, using filtered seawater and fixed in buffered 4% formaldehyde solution. Sections were sorted under x12 and x20 magnification. Macrofauna were identified to phylum/sub-phylum and polychaetes to the lowest taxonomic level i.e. genus or species and abundances were recorded. Macrofauna were rinsed in Milli Q water placed in tin cups and dried at 60°C. Organisms containing calcareous parts were decalcified in double boated silver cups with 2 M HCl and dried as above. Cores for sediment isotopes were lyophilised prior to analysis. Lyophilised sediments were decarbonated by addition of excess 1 M HCl, incubated for 24 h at 30°C in an acid-fumed environment and dried to constant weight.

Macrofaunal and sediment total C and N contents and isotopic ratios were determined using a Flash EA 1112 Series Elemental Analyser connected via a Conflo III to a Delta^Plus^ XP isotope ratio mass spectrometer (Thermo Finnigan). Isotope ratios were calculated with respect to CO_2_ and N_2_ reference gases injected with each sample. Isotopic values of gases were directly referenced against IAEA reference materials USGS40 and USGS41 (both _L-_glutamic acid), both certified for δ^13^C (%VPDB) and δ^15^N (%air N_2_). The C and N content of the samples were calculated from the area output of the mass spectrometer calibrated against the National Institute of Standards and Technology (NIST) standard reference material 1547 peach leaves, which was analysed with every batch of ten samples. Long-term isotope measurement precisions relative to a quality control standard (milled flour) were: total C = 40.3±0.42%, δ^13^C = −25.5±0.29‰, total N = 1.7±0.04% and δ^15^N = 0.367±0.0002‰ (mean ± SD, n = 200). Isotope ratio data were expressed in δ units (‰) and used to estimate faunal ^13^C and ^15^N uptake and incorporation.

Owing to natural variation observed in the natural abundance stable isotopic data in both this study and that of Sweetman & Witte [30], fauna from the experiments were considered to be enriched in ^13^C and ^15^N when their isotopic composition was >−14‰ and >20‰, respectively. Nematodes and foraminifera had high natural δ^15^N values and were considered to be enriched in ^15^N if their isotopic composition was >25‰. Foraminifera also had isotopically heavy natural δ^13^C values and were therefore considered to be enriched in ^13^C when their signatures were >−5‰.

Enrichment of ^13^C or ^15^N in macrofauna was calculated as excess above natural abundance levels and is expressed as specific uptake: Δδ = (δ_sample_ – δ_background_). Specific uptake is a qualitative measure of label uptake based on isotope ratio data. Incorporation of ^13^C and ^15^N into faunal biomass is a quantitative measure [23] and was calculated as the product of the excess atom % of ^13^C or ^15^N (difference in atom %^13^C or ^15^N between sample and background) and C or N content (expresses as unit weight): ^13^C or ^15^N incorporation (unit wt^13^C or ^15^N) = (atom% ^13^C_sample_ or ^15^N_sample_ – atom % ^13^C_background_ or ^15^N_background_) × (unit wt C or N of organism). Incorporation of ^13^C or ^15^N was then adjusted to account for algal labeling, yielding total C or N uptake: uptake (unit wt C or N) = ^13^C or ^15^N incorporation/15 atom % (for ^13^C labelled diatoms) or 7 atom % (for ^15^N labelled coccolithophores). The data used in the incorporation and biomass specific calculations are given in Tables S1 to S3.

Unfortunately, δ^15^N analysis was not conducted on the polychaetes from Exp. 2. Only the elemental C and δ^13^C values were determined on fauna from September.

### Data analyses

The multivariate community data on major groups (standardized fourth root transformed, Bray-Curtis similarity was used to calculate resemblance) was analysed by means of non-parametric permutational (9999 permutation) ANOVA (PERMANOVA), [47], [48] to assess differences between controls and experimental cores, between seasons and between sediment layers. The data set was analysed using a 3-factor mixed model design factors: Treatment (experiment or control) - fixed Season - fixed and Sediment Depth - fixed in PERMANOVA+ for PRIMER. For the experimental data each organism analysed represented a data point in the matrices.

Isotope data from the June experiments were analysed in PERMANOVA. Models were run on normalized fourth root transformed, Euclidean distance similarity matrices. The δ^13^C and δ^15^N tracer incorporation data from the two experiments (Exp. 1 _diatom_C and _cocco_N; Exp.2 _diatom_C and _diatom_N) was analysed by using mixed model designs to assess (1) if there were differences in incorporation of C and N between Exps.1 and 2, (2) if there were differences in the incorporation of C and N between taxa and (3) if there were differences in incorporation between sediment layers. Model factors include: Experiment - fixed, Taxonomic group - fixed and Sediment Depth - fixed. The taxonomic groups analysed in the model included: Foraminifera, Nematoda and Crustacea. The data were classified into two sediment layers: 0 to 2 cm and 2 to 5 cm.

The response of the polychaetes to the food choice experiments were examined by means of a 2 factor mixed model to assess (1) if there were differences in the incorporation of C and N at the family level (2) if there were differences in incorporation between sediment layers. Model factors include: Polychaete family - fixed and Sediment Depth - fixed. Differences between polychaete feeding type - fixed were analysed separately in a 1-factor model with unrestricted permutation of the raw data. Differences in polychaete δ^13^C tracer incorporation between the two experiments were analysed by means of a 1-factor model, experiment - fixed with unrestricted permutation of the raw data.

The same data analysis procedures were carried out in PERMANOVA on both the biomass specific incorporation results and on the natural abundance C∶N ratios, δ^13^C and δ^15^N values of the fauna from the control cores.

Univariate data, e.g. sediment ^13^C values and incorporation of diatom carbon during the September experiments were tested for normality and eveness (Shapiro-Wilk's and Levene's tests, respectively). If the data met these assumptions differences between factors e.g. taxon or sediment depth were tested for using a one-way analysis of variance was used (ANOVA) and if the data were not normally or evenly distributed then a Kruskal-Wallis or a Mann-Whitney *U*-test was applied.

## Results

### Macrofaunal assemblage

**Figure 1:**
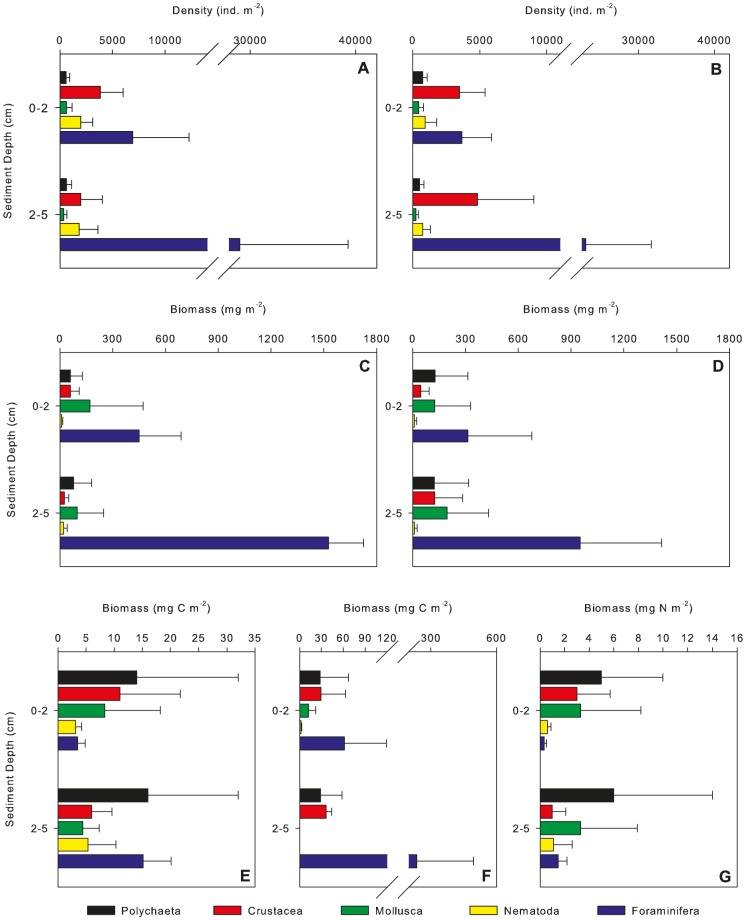
Vertical distribution of: macrofaunal metazoan abundance during (a) June and (b) September; macrofaunal biomass (mg dry weight m^−2^) during (c) June and (d) September; macrofaunal biomass (mg C m^−2^) during (e) June and (f) September; and macrofaunal biomass (mg N m^−2^) during (g) June. Bars represent means ±1 standard deviation, n = 20 for June and 7 for September.

Description of the macrofaunal community structure at Station M was based upon specimens recovered from both background and experimental cores (n = 20 for June and n = 7 for September) and each core was treated as a single replicate. There was no significant difference in the macrofaunal density, biomass dry weight or biomass C normalised to m^2^ between background cores and the experimental cores from each spreader (Table 2). There was no significant difference in density (ind. m^−2^) between seasons (Fig. 1a & 1b, Table 2). There were significant differences in density between sediment depths (Fig. 1a & 1b, Table 2). Simper analyses revealed, foraminifera and crustaceans were responsible for ∼38% and 19% of the variation in density between sediment depths, respectively. The majority (∼80%) of foraminifera were located at 2–5 cm, whilst 58% of crustaceans were located in the upper 2 cm of the sediment (Fig. 1a & 1b). Macrofaunal biomass (dry weight mg m^−2^) did vary significantly between both seasons and horizons (Fig. 1c &1d, Table 2). Foraminifera and molluscs accounted for 32% and 26% of the seasonal variation and 34% and 25% of the variation between sediment depths. There were no significant differences in biomass C between seasons or sediment depths (Fig. 1e & 1f, Table 2).

**Table 2:** Results from multivariate 3 factorial PERMANOVA analyses.

Factor	Density	Biomass	Biomass C
	p value	*pseudo-F*_1,46_	p value	*pseudo-F*_1,29_	p value	*pseudo-F*_1,25_
Treatment	0.117	2.2778	0.053	3.2267	0.1755	1.6925
Season	0.0812	0.0812	0.0268	4.0815	0.1175	2.0502
Sediment depth	0.0002	10.52	0.0335	3.6924	0.1976	1.617

Differences in density (ind. m^−2^), biomass (mg m^−2^ dry weight) and biomass C (mg C m^−2^) between core types, season and sediment depth.

The foraminifera were numerically dominant representing >70% of the community. The majority of foraminifera in the samples were agglutinated and calcareous species e.g. *Globobulimina* and *Cyclammina*. The nematodes represented <10% of the total macrofaunal community. Crustaceans were the dominant metazoan macrofaunal group followed by the polychaetes accounting for >70% and ∼15%, of the metazoan density respectively. Conversely, the polychaetes were the dominant taxon in terms of biomass C and N (Fig. 1e, &1g).

**Figure 2:**
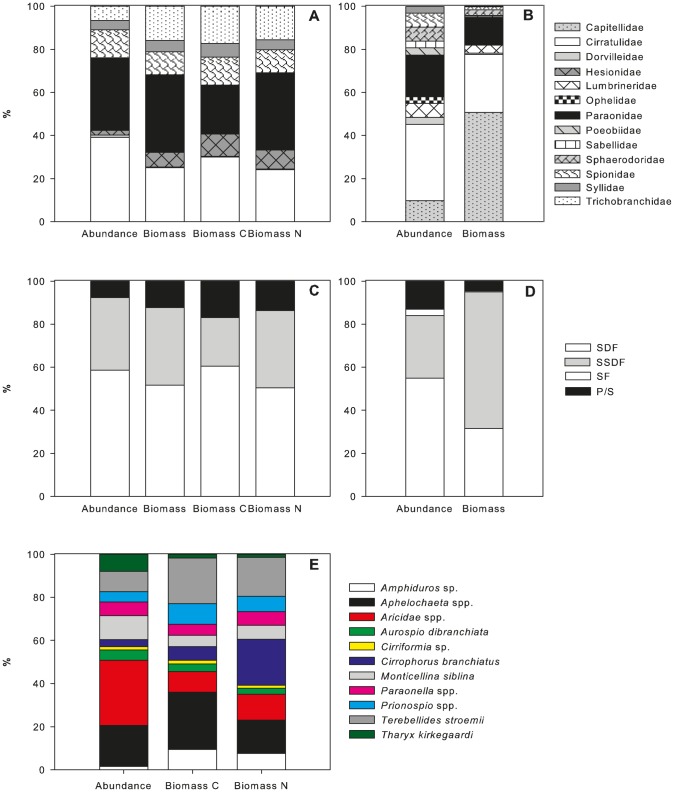
Relative proportions of abundance, biomass (dry weight), biomass C and biomass N of polychaete families in (a) June and (b) September. Relative proportions of abundance, biomass (dry weight), biomass C and biomass N of polychaete feeding types in (c) June and (d) September. Relative proportions of abundance, biomass C and biomass N of polychaete species in (e) June.

A total of 23 polychaete species were identified from the June experiments. Polychaete families were numerically dominated by the Cirratulidae and Paraonidae (Fig. 2a & 2b). Dominant species within the cirratulids were *Aphelochaeta* spp. and *Monticellina siblina* (Fig. 2e). The dominant genus within the paraonids was *Aricidea* spp.. In terms of biomass cirratulids and paraonids were still important but a few families with large-sized individuals became significant contributors to total biomass C and N e.g. Trichobranchidae and Hesionidae (Fig. 2a).

Polychaetes were classified into feeding types according to Fauchald and Jumars [49], and were dominated by surface deposit feeders (SDF; Fig. 2c & 2d) and subsurface deposit feeders (SSDF). In terms of biomass C and N SDF were the most important group followed by SSDF. Surface deposit feeding polychaetes were represented by the cirratulids, spionids and trichobranchids. The subsurface deposit feeders contained paraonids and capitellids. Predator/scavengers included hesionids, dorvilleids, lumbrinerids and syllids.

### Background isotope levels and food web structure

**Figure 3:**
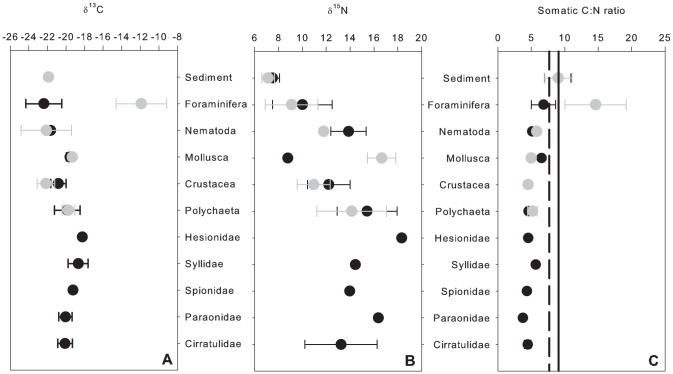
Natural stable isotopic signatures of sediments and macrofauna at Station M, (a) δ^13^C in ‰ (b) δ^15^N in ‰ and (c) C:N ratios, dotted line represents C∶N ratio of POM flux at 50 m.a.b. and solid line represents C:N ratio of surface sediments. Data are means (±1 standard deviation) from a series of 8 pushcores. For the sediments and foraminifera black symbols represent surface samples (0–2 cm) and grey symbols represent sub-surface samples (2–5 cm).

The natural abundance δ^13^C, δ^15^N values and C∶N ratios of sediments and fauna are summarised in Fig. 3. No significant difference in the isotopic composition of the sediments was observed between depths (Figs. 3a, 3b; δ^13^C Mann-Whitney *U* test: p = 0.245; δ^15^N *F_1,6_* = 0.801, p = 0.405). There was no significant difference in faunal δ^13^C composition between June and September (Mann-Whitney *U*-test: p = 0.981) and so data in Fig. 3a include δ^13^C values of fauna from both June and September. Faunal carbon and nitrogen isotopes exhibited wide ranges in values, particularly within the foraminifera. The majority of consumers were enriched in ^13^C and ^15^N compared to surficial sediments (Fig. 3a, 3b).

Values of δ^13^C were significantly different between taxonomic group and sediment depth and these factors interacted (Fig. 3a, Table 3). Pairwise comparisons (Table S4) revealed that polychaetes were significantly enriched in ^13^C compared to crustaceans and nematodes in both sediment depths i.e. 0–2 cm and 2–5 cm. Foraminiferans were significantly enriched in ^13^C compared to all taxa in the 2–5 cm sediment layer. Foraminifera in surface sediments were significantly depleted in ^13^C compared to deeper sediments (PERMANOVA p = 0.002, t = 9.39, unique perms = 700). Deeper living crustaceans were significantly depleted in ^13^C compared to surface dwellers (p = 0.001, t = 9.3932, unique perms = 1287).

**Table 3:** Results from multivariate 2 factorial PERMANOVA analyses.

Factor	δ^13^C	δ^15^N	C/N
	p value	*pseudo-F*_4,50_	p value	*pseudo-F*_4,35_	p value	*pseudo-F*_4,28_
Taxonomic group	0.001	26.979	0.00225	3.3417	0.0001	12.948
Sediment depth	0.0014	16.303	0.00226	6.3059	0.0181	6.9236
TG vs. SD	0.0001	46.561	0.7744	0.44278	0.0947	2.2668

Differences in δ^13^C and δ^15^N signatures and C/N ratios between taxonomic groups (TG) and sediment depths (SD).

There were significant differences in δ^15^N values between taxonomic groups and sediment depth (Fig. 3b, Table 3.) Pairwise comparisons revealed that polychaetes were significantly enriched in ^15^N compared to crustaceans and foraminifera. Fauna found deeper in the sediment were significantly lighter compared to those in the upper 2 cm (mean δ^15^N = 13.4±3.9‰ for 0–2 cm and 11.4±3.0‰ for 2–5 cm). There were significant differences in C∶N ratio between taxonomic groups and sediment depth (Fig. 3c, Table 3). Pairwise comparisons revealed that foraminifera had significantly higher C∶N ratios than all other taxa (Table S5). Fauna in the upper 2 cm of the sediment had lower C∶N ratios than those living deeper (mean C∶N = 4.7±1.5 for 0–2 cm and 7.9±5.2 for 2–5 cm).

### Macrofaunal response to different food sources

#### Incorporation of phytodetrital C and N

**Figure 4:**
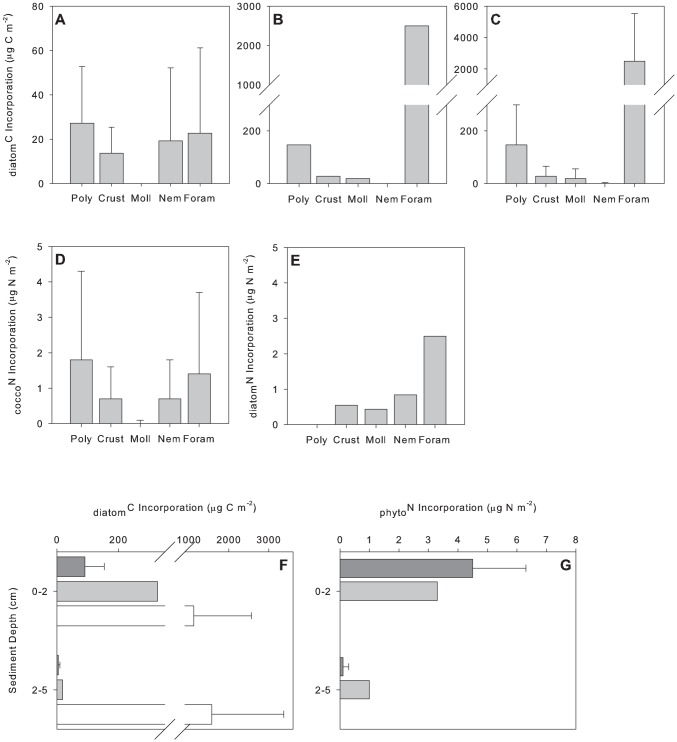
Mean incorporation µg C m^−2^ or µg N m^−2^ (±1 standard deviation) of (a) _diatom_C from Exp. 1 in June; (b) _diatom_C from Exp. 2 in June; (c) _diatom_C from September; (d) _cocco_N from Exp. 1; (e) _diatom_N from Exp. 2. Vertical distribution of (f) _phyto_C and (g) _phyto_N. In plots f and g, dark grey bars represent incorporation from Exp. 1, light grey bars represent incorporation from Exp. 2 and white bars represent mean incorporation of tracer from September. Note the data presented from Exp. 2 in June represents total incorporation µg C m^−2^ or µg N m^−2^. Number of replicates in Exp. 1 = 3 and Exp. 2 = 1 and September  = 3.

Rapid ingestion of label occurred in all experiments. During June in Exp. 1∼25% and 20% of the animals analysed had incorporated ^13^C and ^15^N, respectively. In Exp. 2∼52% and 42% of the animals analysed had incorporated ^13^C and ^15^N, respectively. During September ∼49% of the animals analysed had incorporated ^13^C. No significant difference in label incorporation between taxonomic groups (foraminifera, nematoda and crustacea) was evident (Fig. 4, for June see Table 4; for September including all groups, Kruskal-Wallis: p = 0.218). Significant differences were noted between sediment depths in June (Table 4), >75% of algal carbon and nitrogen was incorporated in the upper 2 cm of the sediment (Fig. 4f–g). No significant difference in algal carbon incorporation was noted in September (Mann-Whitney *U*-test: p = 0.067, Fig. 4f–g). There was no significant difference in incorporation of diatom carbon between June (Exp. 2) and September (Mann-Whitney *U*-test: p = 0.413; Fig. 4b & 4c).

**Table 4:** Results from multivariate PERMANOVA analyses.

Factor	Exp. 1 and 2	Exp 1	Polychaetes
	p value	*pseudo-F*_2,49_	p value	*pseudo-F*_4,45_	p value	*pseudo-F*_1,21_
Experiment	0.7298	0.31504	-	-	0.0046	4.4408
Taxonomic group	0.2977	1.2663	0.4655	0.95774		
Sediment depth	0.0034	7.1032	0.0045	6.3689		

Differences in incorporation of ^13^C and ^15^N labelled phytodetritus in Exp. 1 and 2 between: experiments, taxonomic groups and sediment depths. Differences in incorporation of ^13^C labelled diatoms and ^15^N labelled coccolithophores in Exp. 1 between: taxonomic groups and sediment depths. Differences in incorporation of ^13^C labelled diatoms between polychaetes in Exp 1 and 2.

The PERMANOVA model results (based on label incorporation in crustaceans, nematodes and foraminiferans) indicated no significant difference between the two experiments in June (Fig. 4a, b, d&e Table 4). There was no correlation between the incorporation of _diatom_C and _cocco_N in Exp. 1 (r_s_ = 0.957, p = 0.05) or between _diatom_C and _diatom_N in Exp. 2 (r_s_ = 0.393, p = 0.05). Consequently, in Exp. 1, there was no significant difference between the proportion of _diatom_C and _cocco_N incorporated by the macrofaunal community as a whole (C = 0.012±0.004% and N = 0.008±0.001%; *F_1,4_* = 2.581, p = 0.183).

Taking into account all taxa, in Exp. 1 incorporation of _diatom_C and _cocco_N was not significantly different between taxonomic groups (Fig. 4a & d, Table 4). Significantly greater amounts of both _diatom_C and _cocco_N (>95%) were assimilated in the upper 2 cm of sediment (Fig. 4f & 4g, Table 4). No significant differences were noted between polychaete families or feeding types (PERMANOVA p = 0.227 and p = 0.8997, respectively).

It was only possible to compare incorporation of _diatom_C by the polychaetes between the two experiments. There were marginal differences in polychaete incorporation of _diatom_C between these two experiments (Fig. 4a & 4b; Table 4). In Exp. 1 *Terebellides stroemii* was responsible for ∼87% of the _diatom_C assimilated by the polychaetes, whilst in Exp. 2. *Aricidea* spp. and *Prionospio* sp. assimilated ∼49% and 48% of _diatom_C, respectively.

#### Biomass specific incorporation of phytodetrital C and N

**Figure 5:**
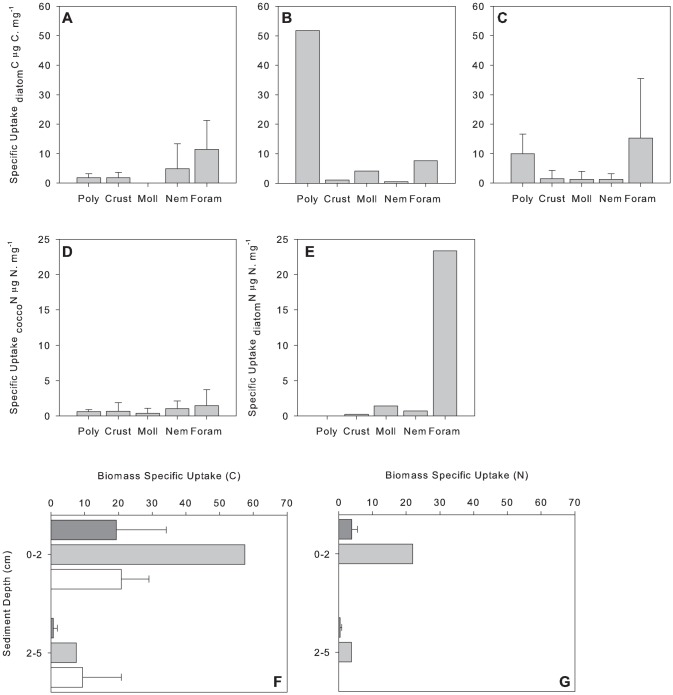
Mean biomass specific uptake µg C mg^−1^ or µg N mg^−1^ (±1 standard deviation) of (a) _diatom_C from Exp. 1 in June; (b) _diatom_C from Exp. 2 in June; (c) _diatom_C from September; (d) _cocco_N from Exp. 1; (e) _diatom_N from Exp. 2. Vertical distribution of biomass specific uptake of (f) _phyto_C and (g) _phyto_N. In plots f and g, dark grey bars represent incorporation from Exp. 1, light grey bars represent incorporation from Exp. 2 and white bars represent mean incorporation of tracer from September. Note the data presented from Exp. 2 in June represents total biomass specific uptake µg C mg^−1^ or µg N mg^−1^. Number of replicates in Exp. 1 = 3 and Exp. 2 = 1 and September  = 3.

The biomass specific incorporation did not differ significantly between Exp. 1 and Exp. 2 in June (Fig. 5, Table 5). However, differences in biomass specific incorporation of phytodetritus between taxonomic groups and also between sediment depths were significant (Fig. 5, Table 5). There was a significant two-way interaction between experiment and taxon (Table 5). Pairwise comparisons revealed that in Exp. 2 Foraminifera had significantly higher biomass specific incorporation of _diatom_C and _diatom_N compared to nematodes (p = 0.034, t = 2.7171) and crustaceans (p = 0.016, t = 2.7236; Fig. 5b, 5e). Foraminifera had significantly higher biomass specific incorporation of _diatom_N (Exp. 2) compared to _cocco_N (Exp. 1: p = 0.039, Fig. 5d & 5e). In Exp. 1 there were no significant differences in biomass specific incorporation of _diatom_C and _cocco_N between taxonomic groups (Fig. 5a & 5d; p = 0.232, t = 1.9953). Biomass specific C incorporation of the polychaetes was not significantly different between experiments, families or feeding types (p = 0.071, p = 0.783 and p = 0.918, respectively, Fig. 5a & 5b). There was no significant difference in biomass specific incorporation of diatom C between June (Exp.2) and September (Mann-Whitney *U*-test: p = 0.801; Fig. 5b & 5c). No significant differences between taxonomic groups were noted in biomass specific incorporation of diatom C in September (Kruskal-Wallis: p = 0.333; Fig. 5c). There was no significant difference in biomass specific incorporation of diatom C between sediment depths in September (Mann-Whitney *U*-test: p = 0.059; Fig. 5f).

**Table 5:** Results from multivariate 3 factorial PERMANOVA analyses.

Factor	Biomass Specific	C/N ratios
	p value	*pseudo-F*_2,49_	p value	*pseudo-F*_4,93_
Experiment	0.6869	0.37233	0.7712	0.22633
Taxonomic group	0.0093	3.9875	0.0001	18.98
Sediment depth	0.0011	9.9486	-	-
EXP vs. TG	0.0026	3.0641	-	-

Differences in biomass specific incorporation of ^13^C and ^15^N labelled phytodetritus in Exp. 1 and 2 between: experiments, taxonomic groups and sediment depths. And results from multivariate 2 factorial PERMANOVA analyses. Differences in somatic C:N ratios of macrofauna between: controls and experiments (EXP) and taxonomic groups (TG).

#### C∶N Stoichiometry

**Figure 6:**
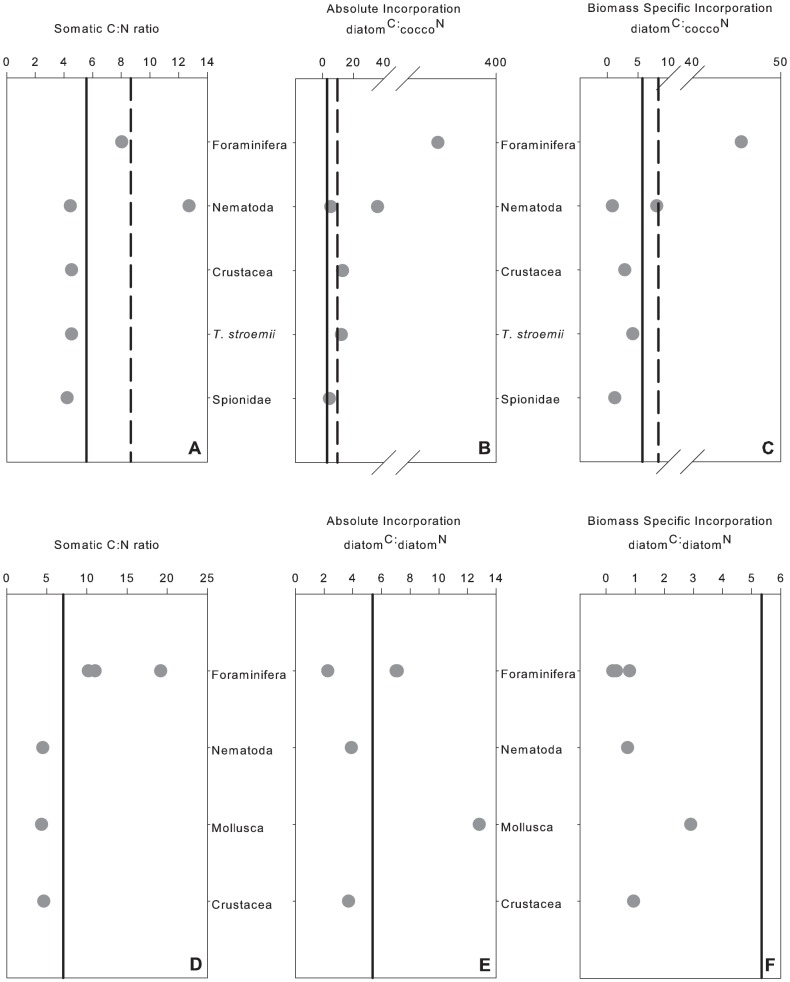
C:N ratios from experimental cores (a) Somatic C:N ratios from Exp. 1; (b) Absolute incorporation C:N ratios from Exp. 1; (c) Biomass specific C:N ratios from Exp.1; (d) Somatic C:N ratios from Exp. 2 (e) Absolute incorporation C:N ratios from Exp. 2. (f) Biomass specific C:N ratios from Exp. 2. Solid black line represent C:N ratios of added diatom tracer and dashed black line represent C:N ratios of added coccolithophorid tracer.

Absolute incorporation C∶N ratios in June (Exps. 1 and 2) ranged from 2.3 to 367 (Fig. 6b & 6e). Biomass specific C∶N ratios ranged from 0.23 to 45.54 (Fig. 6c & 6f). The largest ranges in somatic C∶N ratios were seen in the foraminifera (Fig. 6d). As a result of the small number of organisms that simultaneously ingested ^13^C and ^15^N phytodetritus we were unable to test for significant differences in absolute incorporation and biomass specific C∶N ratios. Somatic C∶N ratios from both control and experimental cores were not significantly different (Table 5). Significant differences were observed in somatic C∶N ratios between taxonomic groups (Table 5). Foraminfera had higher C∶N ratios than all other taxa (Figs. 3c, 6a, 6d; Table 5).

## Discussion

### Macrofaunal assemblage

**Figure 7:**
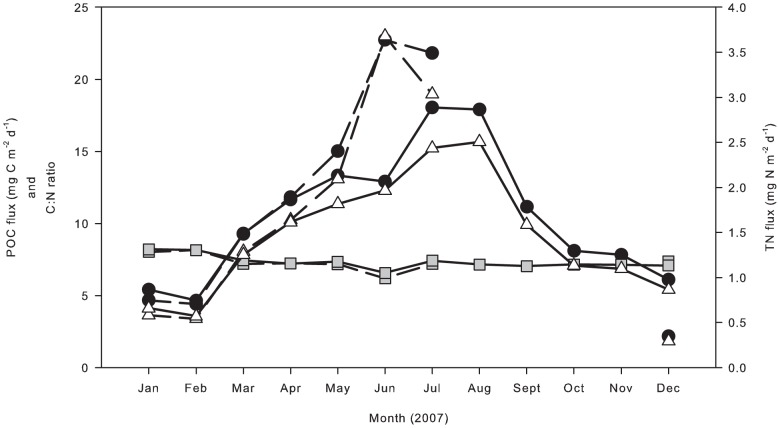
Particulate fluxes at Station M from January to December 2007. Filled circles and open triangles represent the carbon and nitrogen fluxes respectively. Grey-filled squares represent the C/N ratio of flux material. Solid and dashed lines represent flux material from sediment traps moored 600 m.a.b and 50 m.a.b., respectively. There is no data available from the 50 m.a.b. trap from August to November.

Fluxes of POC and PTN to the seafloor were both reaching seasonal highs leading up to and during this study (Fig. 7). Concomitantly, estimates of macrofaunal densities were high but comparable to estimates made by Drazen et al. [20], when particulate fluxes were of a similar magnitude (12 –18 mg C m^−2^ d^−1^ at 600 m.a.b.) and exceeded estimates made by Sweetman and Witte [30] during a period of low particulate flux (∼8 mg C m^−2^ d^−1^ at 600 m.a.b.). The macrofaunal community at Station M during this study may not be food limited as natural food inputs via particulate organic matter flux are at or near the seasonal high. Faunal biomass (relative to C and N) was comparable to estimates for the Whittard canyon and Indian margin [19], [21]; both of the aforementioned study sites are environments sustained by an abundance of organic matter.

Food availability can influence community composition [9]. Community composition (in terms of density) during this study resembled the community described by Drazen et al. [20] and Sweetman and Witte [30]. Switches in the dominant taxonomic groups with respect to biomass were noted in the community composition between periods of high (this study) and low [30] food availability, as well as over longer time scales [9].

The biomass of the foraminifera increased between June and September and significant inputs of POC were observed at Station M from May to September (Fig. 7), which may have been responsible for this increased biomass. Foraminifera are known to respond rapidly to inputs of fresh POC through reproduction and growth [3], [50], [51].

The polychaete community composition is also further evidence of high food availability at Station M during June 2007. The three dominant families, Cirratulidae, Paraonidae and Spionidae are known to be opportunistic and respond quickly to organic enrichment [28], [52].

### Macrofaunal feeding preferences

Macrofaunal stable carbon isotope ratios indicate that the basal food resource is organic C originating from the particulate flux. Macrofaunal δ^13^C values closely mirrored those of the sediments and previous measurements on POC [53]. This can be confirmed by the ingestion of labelled phytodetritus in the experiments.

Faunal incorporation of labelled phytodetritus in all experiments was low <0.005%, and in agreement with previous pulse chase experiments at Station M [30]. At the time of this study Station M did not appear to be food-limited and so the low incorporation of labelled phytodetritus may be a result of satiation of the macrofaunal community or provide evidence of dependence on semi-labile detritus, as demonstrated for the PAP macrofaunal community [54]. The majority of labelled phytodetritus was incorporated in the upper 2 cm of the sediment, in agreement with previous pulse chase studies at abyssal sites and demonstrating that surface deposit feeding community is relatively more important in the processing of OM in abyssal settings than the deeper dwelling fauna [29], [30]. A conspicuous facet of the macrofaunal community at Station M is the opposing zonation of the metazoans and the forminifera. This suggests that the surface dwelling metazoans may play a more substantial role in OM processing at Station M, results from the diatom only treatment support this.

In the diatom treatments incorporation of label by the metazoan macrofauna was an order of magnitude higher compared to the mixed algal treatment. This discrepancy may be a result of experimental design as we were unable to trace the amount of _cocco_C incorporated by the fauna in the mixed algal treatment. Foraminiferal incorporation of carbon was also comparable during both sampling periods. The response of the foraminifera in this study was muted compared to the response observed by Enge et al. [55], at the same site and to foraminifera in other areas e.g. the Indian and Pakistan margins, Sagami Bay [27], [56], [57]. The study of Enge et al. [55] considered foraminifera larger than 250 µm and macrofaunal foraminifera are known to exhibit a retarded response to phytodetritus when compared with smaller foraminifera [58]. Despite the retarded response the proportion of carbon processed by the foraminifera compared to metazoans was high, up to 46% and 90% of the C added in June and September, respectively and is in agreement with previous studies in the N. Atlantic [26], [59]. The majority (>70%) of tracer uptake was observed in foraminifera in the top 2 cm of sediment, this suggests that: (1) surface dwelling foraminifera are important in carbon processing or (2) deeper dwelling foraminifera migrate in the sediment towards the added food source. Migration of foraminifera towards simulated food pulses has been reported previously; by Nomaki et al. [57] and Koho et al. [51]. This seems likely given that the majority of foraminifera in terms of density and biomass were concentrated at 2–5 cm.

Macrofaunal δ^15^N values differed between taxonomic groups indicating that fauna were feeding at different trophic levels and that there is a degree of niche separation between taxon at Station M. Assuming a trophic enrichment factor of 2.2‰ to 3.4‰ [60], [61], foraminifera appear to feed both on relatively depleted POM at the base of the food web and more degraded material. Nematodes and crustaceans were more enriched in ^15^N as has been previously observed at the Porcupine abyssal plain [62], indicating that they either feed at a higher trophic level or on other food sources e.g. degraded POM or bacteria. Crustaceans are known to prey on foraminifera [63]. Whilst, some studies have shown that nematodes have been shown to preferentially feed on bacteria [64], [65], there is also evidence that nematodes do not select for bacterial food sources [66]. Further to this, δ^13^C values of nematodes mirrored those of the sediments, suggesting a sedimentary food source. The enrichment of ^15^N in the nematodes at Station M could result from predation, which has been recently observed in Arctic nematodes [67]. Polychaetes and molluscs were at the apex of the macrofaunal food web at Station M and the associated large ranges in δ^15^N values suggests a degree of omnivory in their diets [68].

There was no difference in incorporation of labelled phytodetritus between the metazoan taxonomic groups during the pulse chase experiments. This is a surprising result given the taxonomic differences in metazoan macrofaunal feeding strategies evident from the natural abundance δ^13^C and δ^15^N values. Closer inspection of the polychaete species in this study reveals a community of highly selective deposit feeders e.g. *Terebellides stroemii*, *Prionospio* spp., *Tharyx kirkegaardi* and *Aricidea* spp.. Evidence from the food web analysis and pulse chase experiments indicates that there is a high degree of plasticity in polychaete feeding strategies at Station M in agreement with the findings of Sweetman and Witte [30].

Dual labelling multiple food sources allows for direct appraisal of selectivity of the macrofaunal community for specific algae when present simultaneously e.g. Herman et al. [69]. As a result of our labelling techniques food sources were significantly enriched in ^13^C and ^15^N compared to non-labelled food sources (Table 2), therefore the isotope mixing model approach used by Herman et al. [61] was not appropriate here. Station M is a food-limited environment, receiving significant food inputs once a year following a spring phytoplankton bloom. We suggest that if the suspected selectivity among algal food sources is indeed important, it may only be evident during certain times. Phytodetritus is usually present at Station M during late Summer and early Autumn. We did not find evidence of metazoan macrofauna selecting for a particular type of phytodetritus at Station M. In our experiments the proportions of _diatom_C and _cocco_N incorporated by the macrofaunal community in the mixed feeding experiments were similar. Futhermore, there was no significant difference in the incorporation of C or N between the mixed feeing experiments or the diatom only treatment and uptake of C and N was not correlated (section 3.3.1.). This suggests that macrofauna do not select for a particular type of phytoplankton when phytodetritus is readily available. Results from the diatom only treatment revealed that metazoans were not incorporating C and N simultaneously (Table S2), suggesting that organism stoichiometry must be considered when interpreting experimental isotope tracer data.

### Macrofaunal C∶N Stoichiometry

Taxonomic differences in C∶N ratios were observed at Station M, foraminifera having the highest C∶N ratios indicating that foraminifera may have a higher demand for C and/or have higher C assimilation efficiencies. This is not a surprising result given that foraminifera grow and reproduce over short temporal scales in the presence of phytodetritus [3], [50], [51]. In this study somatic C∶N ratios of metazoans ranged from 3 to 9, a single nematode had a value of 12. Faunal demands for C and N are driven by energy requirements for somatic growth and reproduction and are balanced against excretion of nitrogenous waste [70]. Somatic tissues of marine invertebrates are dominated by protein and somatic C∶N ratios follow those of amino acids i.e. ranging from 1.5 to 9 [71]. Most marine invertebrates exhibit C∶N ratios ranging from 3 to 8, e.g. [63], [71], [72]. Organisms adapt their feeding strategies in order to maintain nutrient consumption at an optimum level in order to achieve stoichiometric homeostasis [73], [74]. In doing so, the resulting C∶N ratios observed in organisms are often species-specific being regulated by a species physiology [70].

Based on incorporation of tracer, these experiments suggest that faunal demand and/or assimilation of C is higher than for N. In the dual-labelled diatom treatment incorporation of C by metazoans and foraminifera was an order of magnitude higher than for N (∼0.05% of added C was incorporated compared to 0.004% for N). Our results are in agreement with those for the Indian margin, where in an experiment tracing the fate of dual labelled (^13^C and ^15^N) diatoms, more C was processed relative to N [21]. However, the _phyto_C:_phyto_N ratios for biomass incorporation coupled to the somatic C∶N ratios demonstrate a preference for N as has been shown by Hunter et al. [21]. The higher _phyto_C:_phyto_N ratios for absolute assimilation compared to the lower biomass specific ratios may indicate preferential consumption/assimilation of carbon rich molecules such as carbohydrates and lipids as has been previously observed in shallow water corals, calanoid copepods and deep-sea foraminifera [75]–[77]. Furthermore, ammonotelic organisms may rapidly excret _phyto_N resulting in higher absolute assimilation ratios in the fauna [21].

The surface dwelling foraminifera had depleted natural abundance δ^13^C values indicating that they feed at the base of the food web on phytodetrital aggregates. Concomitantly, deeper dwelling foraminifera (2–5 cm) were enriched in ^13^C indicating either preferential utilisation of isotopically light cellular fatty acids during periods of low food availability. Preferential utilisation of isotopically light cellular fatty acids during periods of low food availability (fasting/starvation) can lead to enrichment in ^13^C by up to 4‰ [78]–[80]. Utilisation of cellular fatty acids by deeper dwelling foraminifera would not explain the large differences in δ^13^C values observed here. Large isotopic shifts have been observed in both autotrophic and heterotrophic bacteria [81], [82] and ingestion of bacteria utilizing the reverse tricarboxylic acid pathway can lead to enrichment in ^13^C [83]–[85]. The large ranges in δ^13^C of foraminifera observed in this study are consistent with the ranges for the calcareous and agglutinated foraminifera at Station M during September 2007 [55].

We suggest from the results presented here and previous observations [3], [51], that foraminifera colonize phytodetrital aggregates and use nitrate in respiration at Station M. Foraminifera also had higher biomass specific incorporation of N compared to other taxonomic groups. Consumption of POM is proportional to faunal biomass [86]. At the Indian margin foraminifera have also been shown to incorporate _algal_N in tracer experiments [56]. The foraminifera preferentially selected for diatoms over coccolithophorids and were the only group to show evidence of preferential selection. Foraminifera may require higher levels of organic N from phytodetritus in order to catabolise this food source and fuel intracellular denitrification or to build up reserves of intracellular nitrate for respiration. Recently foraminifera have been shown to carry out complete intracellular denitrification *de novo* and in the presence of endobionts, in a wide range of environments [87]–[89]. Storage of intracellular nitrate within foraminiferal vacuoles is known to result in intracellular δ^15^N_NO3_ values ranging from 12‰ – 42‰ [87], [92]. Foraminiferal δ^15^N values in this study ranged from 6.6‰ to 20.1‰, with three of these values being >12‰, which suggests that some foraminifera at Station M may be accumulating intracellular nitrate.

Nitrate respiration has been observed in phytodetrital aggregates at Station M and was attributed to bacteria [90]. A number of mechanisms for nitrate accumulation in foraminifera have been suggested, these include: transport of nitrate into cells from surrounding porewaters, intracellular production of nitrate, or obtaining nitrate from symbiotic nitrifying bacteria [89]. It is possible that foraminifera at Station M could produce intracellular nitrate via decomposition of _phyto_N to yield NH_4_^+^, which is then converted to nitrate via denitrification either *de novo* or in the presence of bacterial symbionts. However, we are not aware that this process has been observed in foraminifera to date. Alternatively, foraminifera may assimilate nitrate from surrounding porewaters, which could have been produced by decomposition of _phyto_N by bacteria or fauna to yield NH_4_^+^, which is then converted to nitrate by nitrifying bacteria. Koho et al. [91] have demonstrated that foraminifera collect nitrate both in the presence and absence of oxygen, and that foraminifera migrate towards favourable conditions i.e. towards free nitrate and or oxygen. If this is the case at Station M, it might account for the observed foraminiferal distributions. Nitrate storage/respiration in foraminifera has also recently been proposed as a transport mechanism for nitrate in sediments [89], [92]. Our data suggest that foraminifera are important players in nitrogen cycling in abyssal environments and could facilitate the transport of nitrogen through the sediments. However, further work is required to determine the role of foraminifera in both organic and inorganic nitrogen cycling at Station M.

## Supporting Information

Table S1 Isotopic composition of macrofauna recovered from Experiment 1 in June 2007 containing ^13^C-labeled diatoms and ^15^N-labeled coccolithophores.
(DOCX)

Table S2 Isotopic composition of macrofauna recovered from Experiment 2 in June 2007 containing ^13^C and ^15^N- labeled diatoms.
(DOCX)

Table S3 Isotopic composition of macrofauna recovered from the experiment in September 2007 containing ^13^C-labeled diatoms.
(DOCX)

Table S4 Pairwise comparisons of δ^13^C composition between taxonomic groups.
(DOCX)

Table S5 Pairwise comparisons of C:N ratios between taxonomic groups.
(DOCX)
