# Supplementary material for: Feeding Preferences of Abyssal Macrofauna Inferred from In Situ Pulse Chase Experiments
Source: PLoS One. 2013 Nov 26;8(11):e80510. doi: 10.1371/journal.pone.0080510 (PMC3841197; doi:10.1371/journal.pone.0080510)
Supplement: Table S1 — Isotopic composition of macrofauna recovered from Experiment 1 in June 2007 containing 13C-labeled diatoms and 15N-labeled coccolithophores. (DOCX) [file pone.0080510.s001.docx]

**Table S1**

| **Taxon** | **Depth (cm)** | **δ^13^C** | **∆δ^13^C** | **Incorporation** | **Biomass Specific** | **δ^15^N** | **∆δ^15^N** | **Incorporation** | **Biomass Specific** |
| --- | --- | --- | --- | --- | --- | --- | --- | --- | --- |
|  |  |  |  | **µg C m^-2^** | **µg C mg^-1^** |  |  | **µg N m^-2^** | **µg N mg^-1^** |
| **Polychaeta** |  |  |  |  |  |  |  |  |  |
| **Cirratulidae** |  |  |  |  |  |  |  |  |  |
| *Tharyx kirkegaardi* | 0-5cm | -20.0 | .. | .. | .. | 13.3 | .. | .. | .. |
| *Monticellina siblina* | 2-5 cm | -17.4 | .. | .. | .. | 15.7 | .. | .. | .. |
| *Aphelochaeta* sp.1 | 2-5cm | -20.5 | .. | .. | .. | 12.1 | .. | .. | .. |
| unident. | 2-5cm | -19.8 | .. | .. | .. | 13.3 | .. | .. | .. |
| *Aphelochaeta* sp.3 | 3-5cm | -20.1 | .. | .. | .. | 13.1 | .. | .. | .. |
| **Paraonidae** |  |  |  |  |  |  |  |  |  |
| *Aricidea* spp. | 0-3cm | -13.7 | 6.1 | 2.97 | 0.78 | 17.1 | .. | .. | .. |
| unident. | 0-3cm | -16.0 | 3.8 | 0.99 | 0.31 | 17.3 | .. | .. | .. |
| *Cirrophorus branchiatus* | 3-5cm | -19.5 | .. | .. | .. | 18.7 | .. | .. | .. |
| *Paraonella* sp.3 | 3-5cm | -20.0 | .. | .. | .. | 18.9 | .. | .. | .. |
| **Spionidae** |  |  |  |  |  |  |  |  |  |
| unident. | 0-2cm | 8.0 | 27.2 | 4.7 | 1.67 | 53.5 | 39.5 | 1.14 | 1.39 |
| *Aurospio dibranchiata* | 0-5cm | -19.5 | .. | .. | .. | 16.3 | .. | .. | .. |
| **Trichobranchidae** |  |  |  |  |  |  |  |  |  |
| *Terebellides stroemii* | 0-1 cm | -19.4 | .. | .. | .. | 17.2 | .. | .. | .. |
| *Terebellides stroemii* | 0-1 cm | -13.3 | 6.5 | 9.9 | 0.38 | 16.8 | .. | .. | .. |
| *Terebellides stroemii* | 0-1 cm | 30.2 | 50.0 | 52.2 | 2 | 40.3 | 26.3 | 4.27 | 0.48 |
| *Terebellides stroemii* | 1-2 cm | -13.7 | 6.1 | 10.7 | 1.41 | 17.4 | .. | .. | .. |
| *Terebellides stroemii* | 1-2 cm | -19.4 | .. | .. | .. | 17.4 | .. | .. | .. |
| **Crustacea** |  |  |  |  |  |  |  |  |  |
| Replicate 1 | 0-1cm | -18.5 | .. | .. | .. | 16.0 | .. | .. | .. |
| Replicate 1 | 1-2cm | -19.6 | .. | .. | .. | 12.9 | .. | .. | .. |
| Replicate 1 | 2-3cm | -23.0 | .. | .. | .. | 9.3 | .. | .. | .. |
| Replicate 1 | 3-5cm | -24.0 | .. | .. | .. | 9.0 | .. | .. | .. |
| Replicate 2 | 0-1cm | -20.0 | .. | .. | .. | 14.1 | .. | .. | .. |
| Replicate 2 | 1-2cm | 25.5 | 47.0 | 20.7 | 3.49 | 35.1 | 23.3 | 1.59 | 1.22 |
| Replicate 2 | 2-3cm | -17.1 | .. | .. | .. | 27.3 | 15.5 | 0.11 | 0.85 |
| Replicate 2 | 3-5cm | -22.3 | .. | .. | .. | 14.1 | .. | .. | .. |
| Replicate 3 | 0-1cm | -19.8 | .. | .. | .. | 20.3 | 8.5 | 0.38 | 0.16 |
| Replicate 3 | 0-1cm | -19.5 | .. | .. | .. | 15.4 | .. | .. | .. |
| Replicate 3 | 0-1cm | -8.8 | 12.7 | 20.1 | 1.93 | 17.6 | .. | .. | .. |
| Replicate 3 | 1-2cm | -21.1 | .. | .. | .. | 10.7 | .. | .. | .. |
| Replicate 3 | 2-3cm | -22.2 | .. | .. | .. | 8.9 | .. | .. | .. |
| Replicate 3 | 3-5cm | -22.9 | .. | .. | .. | 13.1 | .. | .. | .. |
| **Mollusca** |  |  |  |  |  |  |  |  |  |
| Replicate 1 | 0-2cm | -22.5 | .. | .. | .. | 28.0 | 14.0 | 0.13 | 1.18 |
| Replicate 1 | 2-5cm | -18.4 | .. | .. | .. | 16.5 | .. | .. | .. |
| Replicate 2 | 0-2cm | -19.1 | .. | .. | .. | 15.2 | .. | .. | .. |
| Replicate 2 | 2-5cm | -20.0 | .. | .. | .. | 15.8 | .. | .. | .. |
| Replicate 3 | 0-1cm | n.d. | n.d. | n.d. | n.d. | 18.5 | .. | .. | .. |
| Replicate 3 | 1-2cm | -18.4 | .. | .. | .. | 16.3 | .. | .. | .. |
| Replicate 3 | 3-5cm | n.d. | n.d. | n.d. | n.d. | 16.9 | .. | .. | .. |
| **Nematoda** |  |  |  |  |  |  |  |  |  |
| Replicate 1 | 0-2cm | -21.8 | .. | .. | .. | 32.3 | 19.5 | 0.18 | 1 |
| Replicate 1 | 2-5cm | -20.4 | .. | .. | .. | 20.6 | .. | .. | .. |
| Replicate 2 | 0-2cm | -21.3 | .. | .. | .. | 16.7 | .. | .. | .. |
| Replicate 2 | 2-5cm | -24.9 | .. | .. | .. | 13.5 | .. | .. | .. |
| Replicate 3 | 0-2cm | 171.2 | 193.0 | 55.4 | 14.28 | 46.8 | 33.9 | 1.54 | 1.77 |
| Replicate 3 | 2-3cm | 3.4 | 25.3 | 2.0 | 0.3 | 98.9 | 86.1 | 0.39 | 0.37 |
| Replicate 3 | 3-5cm | -20.2 | .. | .. | .. | 19.1 | .. | .. | .. |
| **Foraminifera** |  |  |  |  |  |  |  |  |  |
| Replicate 1 | 0-1cm | 165.6 | 188.4 | 15.5 | 13.94 | 161.7 | 152.2 | 0.19 | 0.37 |
| Replicate 1 | 1-2cm | 0.5 | 23.2 | 9.6 | 1.73 | 7.4 | .. | .. | .. |
| Replicate 1 | 2-3cm | -21.4 | .. | .. | .. | 9.8 | .. | .. | .. |
| Replicate 1 | 3-5cm | -18.7 | .. | .. | .. | 9.7 | .. | .. | .. |
| Replicate 2 | 0-1cm | -20.3 | .. | .. | .. | 7.9 | .. | .. | .. |
| Replicate 2 | 0-3cm | -20.4 | .. | .. | .. | -0.4 | .. | .. | .. |
| Replicate 2 | 2-3cm | -28.6 | .. | .. | .. | 17.2 | .. | .. | .. |
| Replicate 2 | 3-5cm | -13.7 | .. | .. | .. | 7.9 | .. | .. | .. |
| Replicate 3 | 0-1cm | 206.1 | 228.8 | 69.7 | 16.97 | 165.1 | 155.6 | 4.09 | 4.05 |
| Replicate 3 | 2-3cm | -3.7 | 19.0 | 5.9 | 1.42 | 7.4 | .. | .. | .. |
| Replicate 3 | 3-5cm | -16.9 | .. | .. | .. | 9.0 | .. | .. | .. |
|  |  |  |  |  |  |  |  |  |  |
