# Supplementary material for: Feeding Preferences of Abyssal Macrofauna Inferred from In Situ Pulse Chase Experiments
Source: PLoS One. 2013 Nov 26;8(11):e80510. doi: 10.1371/journal.pone.0080510 (PMC3841197; doi:10.1371/journal.pone.0080510)
Supplement: Table S2 — Isotopic composition of macrofauna recovered from Experiment 2 in June 2007 containing 13C and 15N- labeled diatoms. (DOCX) [file pone.0080510.s002.docx]

**Table S2**

| **Taxon** | **Depth (cm)** | **δ^13^C** | **∆δ^13^C** | **Incorporation** | **Biomass Specific** | **δ^15^N** | **∆δ^15^N** | **Incorporation** | **Biomass Specific** |
| --- | --- | --- | --- | --- | --- | --- | --- | --- | --- |
|  |  |  |  | **µg C m^-2^** | **µg C mg^-1^** |  |  | **µg N m^-2^** | **µg N mg^-1^** |
| **Polychaeta** |  |  |  |  |  |  |  |  |  |
| **Cirratulidae** |  |  |  |  |  |  |  |  |  |
| *Tharyx kirkegaardi* | not avail. | 108.2 | 128.0 | 6.6 | 0.72 | n.d. | n.d. | n.d. | n.d. |
| **Paraonidae** |  |  |  |  |  |  |  |  |  |
| *Aricidea* spp. | not avail. | 472.8 | 492.6 | 156.3 | 40.81 | n.d. | n.d. | n.d. | n.d. |
| *Aricidea simplex* | not avail. | -19.6 | .. | .. | .. | n.d. | n.d. | n.d. | n.d. |
| *Paraonella* spp. | not avail. | -2.3 | 17.5 | 0.9 | 0.14 | n.d. | n.d. | n.d. | n.d. |
| **Spionidae** |  |  |  |  |  |  |  |  |  |
| *Prionospio* sp.2 | not avail. | 294.6 | 313.8 | 153.5 | 9.96 | n.d. | n.d. | n.d. | n.d. |
| unident. | not avail. | -12.9 | 6.3 | 0.2 | 0.08 | n.d. | n.d. | n.d. | n.d. |
| **Crustacea** |  |  |  |  |  |  |  |  |  |
| Replicate 1 | 0-1cm | -19.6 | .. | .. | .. | 12.4 | .. | .. | .. |
| Replicate 1 | 0-1cm | -1.0 | 20.6 | 1.7 | 0.22 | 28.1 | 16.3 | 0.46 | 0.24 |
| Replicate 1 | 0-1cm | 6.9 | 28.5 | 6.0 | 0.8 | 10.5 | .. | .. | .. |
| Replicate 1 | 1-2cm | -20.6 | .. | .. | .. | 15.2 | .. | .. | .. |
| Replicate 1 | 2-3cm | -22.9 | .. | .. | .. | 21.9 | 10.1 | 0.09 | 0.05 |
| Replicate 1 | 3-5cm | -23.0 | .. | .. | .. | 13.4 | .. | .. | .. |
| **Mollusca** |  |  |  |  |  |  |  |  |  |
| Replicate 1 | 0-5cm | 61.0 | 80.7 | 5.6 | 4.09 | 31.5 | 17.4 | 0.44 | 1.41 |
| **Nematoda** |  |  |  |  |  |  |  |  |  |
| Replicate 1 | 0-1cm | 3.0 | 24.9 | 2.5 | 0.52 | 30.7 | 17.8 | 0.63 | 0.7 |
| Replicate 1 | 1-2cm | -20.5 | .. | .. | .. | 19.5 | .. | .. | .. |
| Replicate 1 | 1-2cm | -19.9 | .. | .. | .. | 37.3 | 24.4 | 0.21 | 0.23 |
| Replicate 1 | 2-3cm | -20.2 | .. | .. | .. | 4.3 | .. | .. | .. |
| Replicate 1 | 3-5cm | -21.5 | .. | .. | .. | 7.7 | .. | .. | .. |
| Replicate 1 | 3-5cm | -19.1 | .. | .. | .. | 17.7 | .. | .. | .. |
| **Foraminifera** |  |  |  |  |  |  |  |  |  |
| Replicate 1 | 0-1cm | 60.3 | 83.0 | 4.2 | 4.2 | 249.0 | 239.5 | 1.85 | 18.46 |
| Replicate 1 | 1-2cm | -5.5 | 17.2 | 0.9 | 0.87 | 40.0 | 30.5 | 0.13 | 2.5 |
| Replicate 1 | 2-3cm | 56.1 | 78.8 | 3.7 | 1.88 | 88.6 | 79.0 | 0.52 | 2.36 |
| Replicate 1 | 2-3cm | 2.0 | 24.8 | 1.3 | 0.66 | 16.9 | .. | .. | .. |
| Replicate 1 | 3-5cm | -18.4 | .. | .. | .. | 10.3 | .. | .. | .. |
| Replicate 1 | 3-5cm | -21.0 | .. | .. | .. | 10.5 | .. | .. | .. |
