# Supplementary material for: Feeding Preferences of Abyssal Macrofauna Inferred from In Situ Pulse Chase Experiments
Source: PLoS One. 2013 Nov 26;8(11):e80510. doi: 10.1371/journal.pone.0080510 (PMC3841197; doi:10.1371/journal.pone.0080510)
Supplement: Table S3 — Isotopic composition of macrofauna recovered from the experiment in September 2007 containing 13C-labeled diatoms. (DOCX) [file pone.0080510.s003.docx]

**Table S3**

| **Taxon** | **Depth (cm)** | **Replicate** | **δ^13^C** | **∆δ^13^C** | **Incorporation** | **Biomass Specific** |
| --- | --- | --- | --- | --- | --- | --- |
|  |  |  |  |  | **µg C m^-2^** | **µg C mg^-1^** |
| **Polychaeta** |  |  |  |  |  |  |
| **Capitellidae** |  |  |  |  |  |  |
| *Capitellethus* sp. | 0-2 cm | 1 | -18.82 | .. | .. | .. |
| *Capitellethus* sp. | 2-5 cm | 1 | -19.88 | .. | .. | .. |
| *Capitellethus* sp. | 0-2 cm | 2 | -16.11 | .. | .. | .. |
|  |  |  |  |  |  |  |
| **Cirratulidae** |  |  |  |  |  |  |
| *Aphelochaeta* sp. | 0-2 cm | 3 | 346.6 | 367.2 | 97.1 | 10.8 |
| unident. | 2-5 cm | 3 | -7.47 | 13.1 | 8.9 | 0.1 |
| *Tharyx kirkegaardi* | 2-5 cm | 3 | -19.51 | .. | .. | .. |
|  |  |  |  |  |  |  |
| **Lumbrineridae** |  |  |  |  |  |  |
| unident. | 2-5 cm | 2 | -18.41 | .. | .. | .. |
| unident. | 2-5 cm | 4 | -19.09 | .. | .. | .. |
|  |  |  |  |  |  |  |
| **Paraonidae** |  |  |  |  |  |  |
| *Aricidea* spp. | 2-5 cm | 1 | -20.38 | .. |  |  |
| *Aricidea* spp. | 0-2 cm | 4 | 437.0 | 458.0 | 41.9 | 14 |
|  |  |  |  |  |  |  |
| **Peobidae** |  |  |  |  |  |  |
| unident. | 2-5 cm | 2 | -17.24 | .. | .. | .. |
|  |  |  |  |  |  |  |
| **Sabellidae** |  |  |  |  |  |  |
| unident. | 2-5 cm | 4 | 10.21 | 30.9 | 10.14 | 5.1 |
|  |  |  |  |  |  |  |
| **Sphaerodorcidae** |  |  |  |  |  |  |
| unident. | 0-2 cm | 1 | 11.10 | 31.8 | 29.35 | 2.7 |
| unident. | 0-2 cm | 1 | 24.73 | 45.4 | 20.21 | 1.8 |
|  |  |  |  |  |  |  |
| **Polychaeta** |  |  |  |  |  |  |
| unident. | 0-2 cm | 1 | -17.65 | .. | .. | .. |
| unident. | 0-2 cm | 2 | -19.52 | .. | .. | .. |
| unident. | 0-2 cm | 4 | 28.80 | 49.5 | 7.91 | 4.0 |
|  |  |  |  |  |  |  |
| **Crustacea** |  |  |  |  |  |  |
| Replicate 1 | 0-2 cm |  | -18.1 | .. | .. | .. |
| Replicate 1 | 2-5 cm |  | -20.0 | .. | .. | .. |
| Replicate 2 | 0-2 cm |  | -12.8 | 7.8 | 79.9 | 5.7 |
| Replicate 2 | 2-5 cm |  | -20.8 | .. | .. | .. |
| Replicate 3 | 0-2 cm |  | 27.9 | 48.4 | 29.4 | 0.3 |
| Replicate 3 | 2-5 cm |  | -19.4 | .. | .. | .. |
| Replicate 4 | 0-2 cm |  | n.d. | .. | .. | .. |
| Replicate 4 | 2-5 cm |  | -18.1 | .. | .. | .. |
|  |  |  |  |  |  |  |
| **Mollusca** |  |  |  |  |  |  |
| Replicate 1 | 0-5cm |  | -14.2 | 5.1 | 73.7 | 5.3 |
| Replicate 2 | 0-5cm |  | -17.9 | .. | .. | .. |
| Replicate 3 | 0-5cm |  | n.d. | .. | .. | .. |
| Replicate 4 | 0-5cm |  | -17.3 | .. | .. | .. |
|  |  |  |  |  |  |  |
| **Nematoda** |  |  |  |  |  |  |
| Replicate 1 | 0-5cm |  | 86.8 | 108.1 | 4.2 | 4.1 |
| Replicate 2 | 0-5cm |  | -9.1 | 12.2 | 1.6 | 0.5 |
| Replicate 3 | 0-5cm |  | n.d. | .. | .. | .. |
| Replicate 4 | 0-5cm |  | n.d. | .. | .. | .. |
|  |  |  |  |  |  |  |
| **Foraminifera** |  |  |  |  |  |  |
| Replicate 1 | 0-5cm |  | 454.3 | 473.6 | 6175 | 42.6 |
| Replicate 2 | 0-2 cm |  | 265.7 | 281.3 | 667.6 | 12.4 |
| Replicate 2 | 2-5 cm |  | 1.4 | 22.5 | 3137 | 6.3 |
| Replicate 3 | 0-5cm |  | -8.9 | .. | .. | .. |
| Replicate 4 | 0-2 cm |  | 3.4 | 18.9 | 32.6 | 0.1 |
| Replicate 4 | 2-5 cm |  | n.d. | .. | .. | .. |
