# Supplementary material for: Feeding Preferences of Abyssal Macrofauna Inferred from In Situ Pulse Chase Experiments
Source: PLoS One. 2013 Nov 26;8(11):e80510. doi: 10.1371/journal.pone.0080510 (PMC3841197; doi:10.1371/journal.pone.0080510)
Supplement: Table S4 — Pairwise comparisons of δ13C composition between taxonomic groups. (DOCX) [file pone.0080510.s004.docx]

**Table S4**

| **Groups** | **t** | **P (perm)** | **Den. df** |
| --- | --- | --- | --- |
| Polychaeta, Crustacea | 7.4816 | 0.001* | 24 |
| Polychaeta, Mollusca | 0.56616 | 0.654 | 12 |
| Polychaeta, Nematoda | 4.4875 | 0.001* | 18 |
| Polychaeta, Foraminifera | 4.9512 | 0.001* | 23 |
| Crustacea, Mollusca | 3.3794 | 0.008* | 12 |
| Crustacea, Nematoda | 0.35483 | 0.745 | 18 |
| Crustacea, Foraminifera | 8.1003 | 0.001* | 23 |
| Mollusca, Nematoda | 1.4948 | 0.272 | 6 |
| Mollusca, Foraminifera | 2.0415 | 0.071 | 11 |
| Nematoda, Foraminifera | 5.7842 | 0.001* | 17 |
