# Supplementary material for: Feeding Preferences of Abyssal Macrofauna Inferred from In Situ Pulse Chase Experiments
Source: PLoS One. 2013 Nov 26;8(11):e80510. doi: 10.1371/journal.pone.0080510 (PMC3841197; doi:10.1371/journal.pone.0080510)
Supplement: Table S5 — Pairwise comparisons of C:N ratios between taxonomic groups. (DOCX) [file pone.0080510.s005.docx]

**Table S5**

| **Groups** | **t** | **P (perm)** | **Den. df** |
| --- | --- | --- | --- |
| Polychaeta, Crustacea | 0.52278 | 0.6622 | 17 |
| Polychaeta, Mollusca | 0.74941 | 0.4946 | 6 |
| Polychaeta, Nematoda | 2.2937 | 0.0639 | 7 |
| Polychaeta, Foraminifera | 6.0012 | 0.0003* | 13 |
| Crustacea, Mollusca | 0.002062 | 0.9982 | 13 |
| Crustacea, Nematoda | 0.80758 | 0.4261 | 14 |
| Crustacea, Foraminifera | 5.5731 | 0.0003* | 20 |
| Mollusca, Nematoda | 0.94649 | 0.4104 | 3 |
| Mollusca, Foraminifera | 3.3664 | 0.0079* | 9 |
| Nematoda, Foraminifera | 3.2944 | 0.0087* | 10 |
